# Synthesis and high-resolution structural and chemical analysis of iron-manganese-oxide core-shell nanocubes

**DOI:** 10.1038/s41598-019-55397-z

**Published:** 2019-12-17

**Authors:** Aladin Ullrich, Mohammad Mostafizar Rahman, Paolo Longo, Siegfried Horn

**Affiliations:** 1 University of Augsburg, Institute of Physics, Universitätsstr. 1, 86159 Augsburg, Germany; 2 Gatan, Inc. 5794, W Las Positas BLVD, Pleasanton, CA 94588 USA

**Keywords:** Nanoparticles, Nanoparticle synthesis

## Abstract

We have investigated the structure and chemical composition of nanoparticles synthesized by thermal decomposition of a mixture of iron oleate and manganese oleate in a high-boiling solvent in the presence of Na-oleate and oleic acid as surfactants by analytical transmission electron microscopy (TEM). The particles appear core-shell like in bright field TEM images. Higher spatial resolution TEM (HRTEM) analysis reveals a FeO/MnO like structure in the core and a spinel like structure in the shell. With high-resolution analytical methods like energy dispersive x-ray spectroscopy (EDS) and electron energy loss spectroscopy (EELS), the distribution of the metals Mn and Fe was investigated. Differences in the oxidation state of these metals were found between the core and the shell region. The presence of sodium from the used surfactant (Na-oleate) on the surface of the particles has been proved.

## Introduction

## Motivation

Transition metal oxide nanoparticles show interesting size- and shape-dependent properties due to their wide diversity in their crystal structure and metal oxidation states. In particular, ferrite nanoparticles can be used in many different fields like sensor technology^1^, various medical applications (hyperthermia, contrast agent, drug delivery, theranostic applications)^2–6^, or water treatment^7,8^. In particular, manganese-ferrite nanoparticles are a potential candidate for hyperthermia due to their biocompatibility and advantageous magnetic properties^2,9^.

The properties of such particles depend strongly on the elemental distribution and surface condition, e.g. on the oxidation state of the metals or on the presence of a core-shell structure. Such core-shell particles with different magnetic phases can show exchange coupling. The magnetism of such particles can be tuned, e.g. the hysteresis behaviour or the specific loss power can be tailored to particular needs by tuning the core-shell structure^10,11^. For medical applications, the biocompatibility may be controlled by the properties of the iron oxide shell^12^.

A widely applied method for the synthesis of such nanoparticles is the thermal decomposition of metal oleate precursors in high boiling solvents like 1-octadecene^13,14^. However, most of the published studies are addressing single metal particles. In this study, the synthesis of bimetal oxide particles by using both Fe-oleate and Mn-oleate at the same time to produce iron-manganese-oxide particles is investigated.

The behaviour of ferritic nanoparticles is discussed controversially in literature. Some researchers observe a complete oxidation of Fe^2+^ containing particles^15,16^, others find stable FeO_x_ cores surrounded by an Fe^3+^ shell^17^. The core-shell particles described in this work appear stable against oxidation for at least one year. The presence of Fe^2+^ in the synthesized particles has been observed and investigated by other authors^18,19^ and was explained by the formation of reducing agents during the synthesis resulting in the reduction of the original Fe^3+^ in the precursor to Fe^2+^ in the particles. The Mn^2+^ ions present after synthesis, on the other hand, seem unaffected.

In the case of bimetal core-shell systems the elemental distribution is crucial for the properties of the particles. For the Fe/Mn system the group of Bodnarchuck *et al*.^20^ found that the decomposition of a mixture of the corresponding oleate precursors leads to core shell particles in which the manganese is only found in the shell. A similar separation of the metal ions during synthesis was observed by Oberdick *et al*.^21^, who used the corresponding metal acetylacetonates as precursors. The different decomposition temperature of the different metal acetylacetonates in combination with a suitable heating profile leads to the formation of iron oxide cores surrounded by manganese oxide. In contradiction, Sun *et al*.^22^ observed homogeneous nanoparticles of the MnFe_2_O_4_ phase when using acetylacetonate precursors. In this work our interest is focused on the elemental distribution and oxidation state of the metal ions in the synthesized particles as well as on the stability of the core-shell system addressing also the above described controversy. To this end we apply TEM, EELS, and EDS.

## Sample Preparation

Particles were synthesized following the method of Park *et al*.^13^ as described below. By this method, monodisperse particles with sizes ranging from 5–25 nm can be produced. The size and shape depends on the synthesis parameters, particularly on the used surfactants. Typical surfactants are oleic acid or sodium oleate. The corresponding metal oleate precursors were synthesized from oleic acid and the corresponding metal chloride salts.

### Precursor preparation

Mn(II)-oleate was produced by reaction of 15 mmol MnCl_2_*4H_2_O with 30 mmol oleic acid in the presence of 30 mmol NaOH in a mixture of 18 ml n-hexane, 10 ml EtOH and 8 ml water. The reaction mixture was heated up and kept at its boiling point of ~60 °C for 4 h in a three neck flask with attached reflux condenser, N_2_-inlet and thermometer. During the whole reaction the mixture was stirred with a magnetic stirrer and heated with a heating mantle. A constant nitrogen gas flow of ~1 ml/s was applied. After 4 h reaction time, the heater was switched off and the solution was allowed to cool down to room temperature overnight while still stirred. The formed pink solid product floating on the top of the solution was taken out of the flask, pestled slightly and washed three times with water and acetone, respectively, on a suction filter. After drying at room temperature, a pale, pinkish powder was obtained.

Fe(III)-oleate was synthesized by the reaction of 10 mmol FeCl_3_*6H_2_O with 30 mmol oleic acid in the presence of 30 mmol NaOH in a mixture of n-hexane, EtOH and water. The reaction parameters were identical to the Mn(II)-oleate except the washing procedure. The iron oleate was separated in a separation funnel and washed three times with 10 ml water. After washing, it was dried under vacuum for 5 h at ~50 °C, until a dark brown, highly viscous liquid was obtained. The so prepared precursors were used within one week after preparation, since further ageing affected the reproducibility of the preparation of the particles.

### Particle preparation

For particle synthesis, the precursors were mixed (ratio Fe:Mn = 2:1) and decomposed in 1-octadecene under nitrogen atmosphere at ~317 °C in the presence of oleic acid and sodium oleate as surfactants. The 1-octadecene was degassed in advance for 30 min at ~50 °C under vacuum to remove oxygen. During this procedure, the flask was vented three times with nitrogen and evacuated again. A Schlenk line was used to avoid air contact during the insertion of the educts in the reaction flask. The setup consisted of a three neck flask with attached reflux condenser, N_2_-inlet, and thermometer. Heating was performed with a heating mantle, stirring with a magnetic stirrer. The reaction solution was heated up to the boiling point of ~317 °C with a heating rate of 25 °C/min. After 30 min boiling, the heating mantle was switched off and the solution was allowed to cool down to room temperature, still under nitrogen atmosphere and permanent stirring.

1 ml of the particle containing dispersion was diluted with 2 ml n-hexane and particles were precipitated by addition of isopropanol. The dispersion was centrifuged, the clear solution poured away and the particles were re-dispersed in 2–3 ml n-hexane. This cleaning procedure was applied three times. For storage, the particles were dispersed in n-hexane. The cleaning procedure and storing was performed under ambient conditions as well as under an inert nitrogen atmosphere (<1 ppm O_2_). Independent of the preparation very similar particles were obtained.

## Characterization

The samples were investigated by (scanning) transmission electron microscopy ((S)TEM), energy dispersive x-ray spectroscopy (TEM-EDS), electron energy loss spectroscopy (TEM-EELS), and x-ray diffraction (XRD). For TEM analysis, the particles were prepared on a conventional carbon coated copper grid. For this purpose, the grid was dipped into the particle dispersion for 1 second and dried under ambient conditions within a few minutes. Standard TEM pictures were taken with a JEOL JEM 2100 F, analytical investigations (EDS and EELS) with a JEOL JEM F200 and a GATAN EELS spectrometer. Both microscopes were operated at 200 keV beam energy. Higher spatial resolution EELS measurements were performed with a GATAN GIF Quantum K2 system with direct detection technology. This system allowed a dwell time of 5 ms and enabled therefore higher spatial resolution mappings with high signal to noise ratio at short timescales without noticeable carbon contamination and sample damage.

## Results

**Figure 1:**
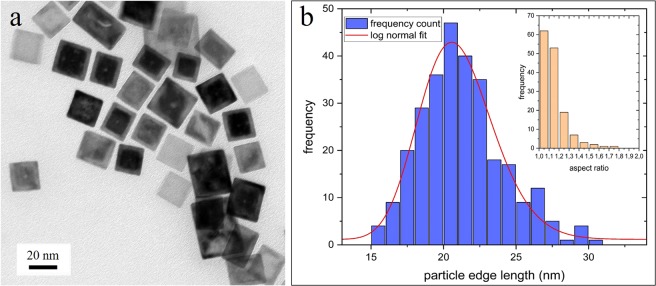
(**a**) Bright field TEM images of the particles (**b**) size distribution with log-normal fit. Inset: aspect ratio plot.

In conventional bright field TEM images the particles appeared cubic shaped, with most particles showing a core-shell like structure, as can be seen in Fig. 1a. The average size (edge length) of the particles is (21 ± 3) nm. A size distribution is shown in Fig. 1b. The core-shell structure is observed for particles processed and stored under ambient conditions as well as for particles processed and stored in a glove box atmosphere without oxygen contact and a nitrogen purge during transfer in the TEM to minimize oxygen contact. The core/shell structure does not change significantly even after an air plasma cleaning treatment applied to several samples before high-resolution STEM investigations. Particles stored for 1 year under ambient conditions still show a comparable core-shell structure with slight increased shell thickness (by about 1 nm).

**Figure 2:**
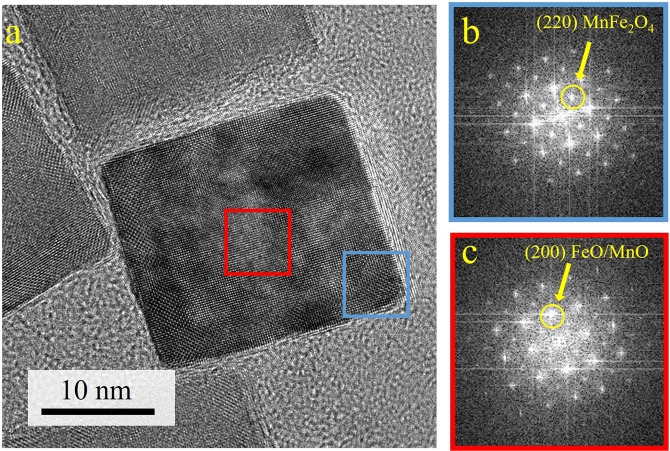
(**a**) High resolution TEM image of a Fe-Mn-oxide nanoparticle close to the [001] orientation of a cubic structure; the FFT from the shell region (**b**) has clear reflections from (220) planes of the spinel structure, while for the central region (**c**) wustite/manganosite structure can be assumed ((220) spinel reflection is almost absent).

Further HR-TEM analysis shows a cubic crystal structure in the shell as well as in the core region, with well matching d-spacings. Almost all particle surfaces show an orientation close to {100} facets of a cubic structure. In Fig. 2, a high resolution TEM image of the core and shell region of a particle is shown using fast Fourier transform (FFT) analysis. The core region shows a diffraction pattern compatible with the wustite/manganosite structure (rock salt structure). The shell shows a similar pattern, but with additional spots compatible with the spinel like structure like magnetite, maghemite or Mn-ferrite. Since the spinel structures of these materials are very similar and indistinguishable by diffraction or HR-TEM, this phase will be labeled generally as “spinel phase” in the following discussion.

The additional spots only visible in the FFT from the shell region can be labeled as {220} of the spinel phase (Fig. 2b). A careful inspection of the FFT of the core region (Fig. 2c) reveals a very weak spot at the same position, which is assumed to result from the thin layer of the spinel phase at the top and bottom of the particle, which is projected in the 2D plane by the measurement.

The presence of these phases is confirmed by XRD measurements, while at the same time the absence of other phases possibly not visible in the TEM (e.g. large contaminations) is proved. It was not possible to evaluate the data in a way leading to a more precise result of the phases. Due to the small coherence volumes of the core and shell, respectively, the signals are broadened and could not be used for e.g. a Rietveld analysis to get a more precise phase composition.

**Figure 3:**
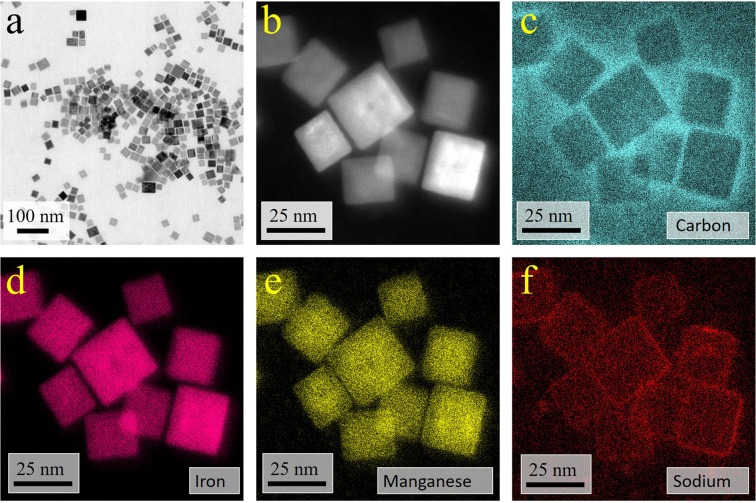
(**a**) BF image of cubic nanoparticles exhibiting core-shell structure; (**b**) DF-STEM-image of the EDS-mapped area; (**c**) carbon map; (**d**) iron map; (**e**) manganese map; (**f**) sodium map.

**Figure 4:**
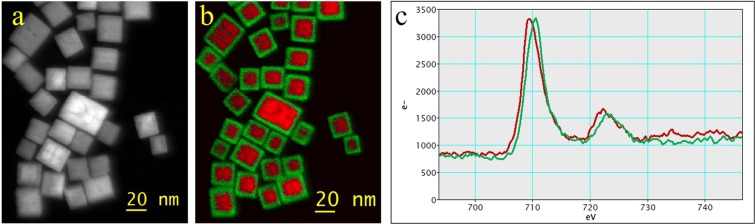
EELS elemental and chemical mappings: (**a**) DF-image; (**b**) Color coded oxidation state map of the iron. Red: Fe^2+^; green: Fe^3+^, according to MLLS fit with (**c**) reference spectra showing typical shift of L_3_-line from Fe^2+^ (red) to Fe^3+^ (green).

The elemental composition of the particles was investigated by high resolution EDS analysis. The corresponding elemental maps are shown in Fig. 3. Although the core-shell structure of the particles is clearly visible in the STEM dark field image (Fig. 3b), no core shell structure is found in the elemental distribution maps. Iron is distributed quite homogeneously within the particles, while the absolute manganese content shows a slight gradient with maximum in the core of the particles. This is consistent with the EELS elemental map of a different particle shown in Fig. 4.

An organic coating originating most likely from oleic acid molecules adsorbed on the surface of the particles is revealed by a carbon signal, that indicates a ~2 nm shell surrounding the particles. Sodium is also expected to be present at the surface, since sodium oleate was used as surfactant during the particle preparation. In Fig. 3f, sodium is clearly visible as thin layer between the surface of the metal oxide particle and the carbon signal. The sodium content in the sample is 2–5 at%, depending on the exact location (single particles vs. cluster region).

**Figure 5:**
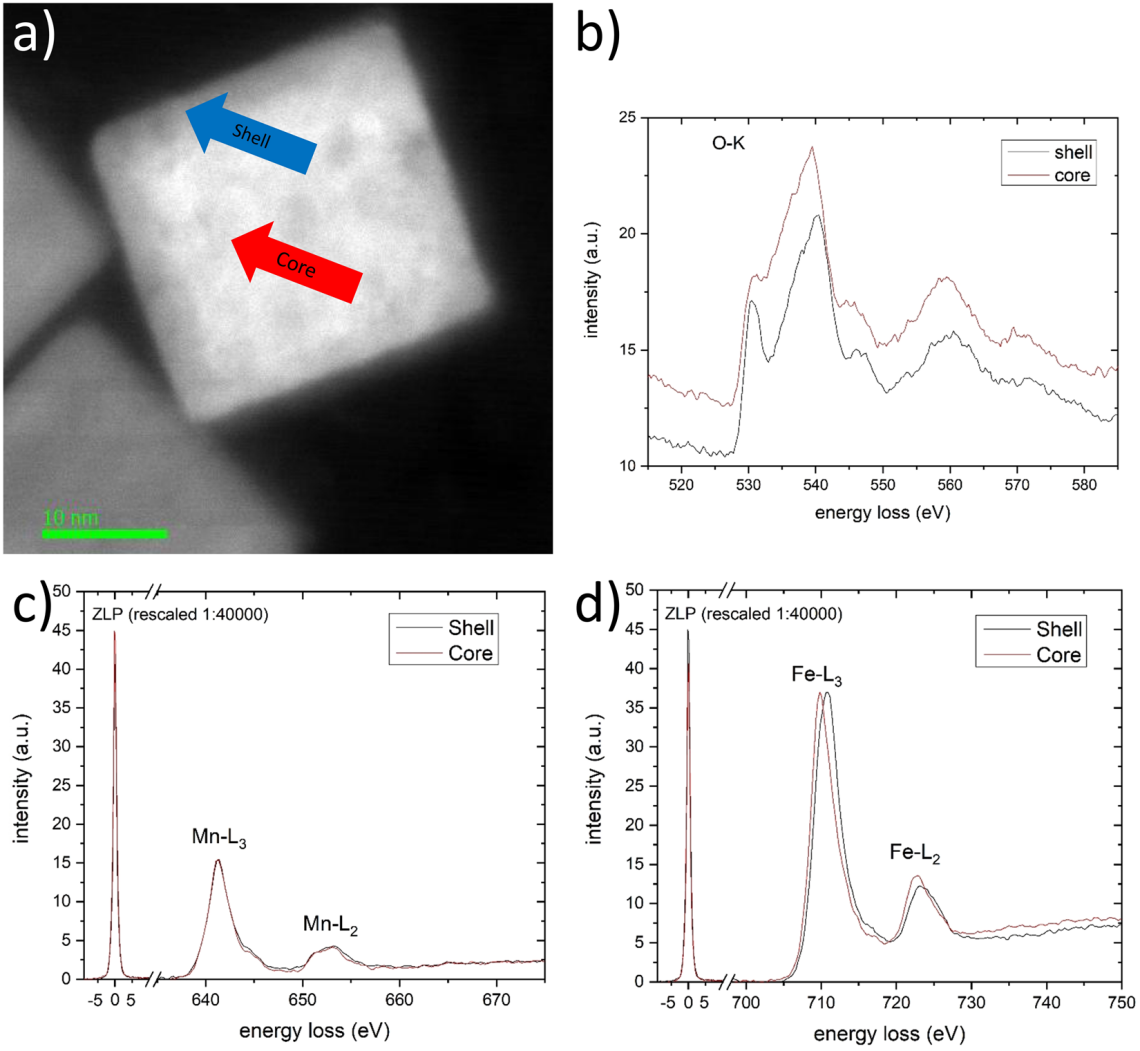
Higher resolution EELS (0.25 eV) measurements of a single nanoparticle. (**a**) DF-STEM image with measured areas indicated by blue and red arrow, respectively; (**b**) Oxygen K edge; (**c**) Mn L edges (normalized) with ZLP; (**d**) Fe K edges (normalized) with ZLP. Spectra are normalized with respect to the metal L_3_ edge.

Since the core-shell structure observed in TEM images is not visible in the EDS elemental maps, we conclude that it arises predominantly due to the Bragg contrast and not due to Z-contrast. To prove this assumption, EELS measurements were performed to determine the oxidation state of the metal ions. Furthermore, different crystal structures can be distinguished by specific fingerprint structures in such spectra. EEL spectra were recorded in the shell and core region. Representative spectra from the core and shell region, respectively, are shown in Fig. 5. The spectra were recorded in DualEELS^TM^ mode, i.e. the low-loss region with the zero loss peak was recorded together with the high-loss region. The zero loss peak was used during data evaluation to calibrate the zero position of all spectra to obtain precise values of the absolute energies of the observed edges.

While for the Mn L_3_ and L_2_ edges, the same energy position is found for both, core and shell region, the iron L_3_ and L_2_ edges show a clear shift of about 0.9 eV towards higher energies from the core region compared to the shell region. In addition to this shift, a slight increase of the peak width of ~7% is observed. This indicates an increase of the oxidation state of iron in the shell compared to the core of the particle^23–25^.

For further analysis the ratio of the L_3_/L_2_ intensity was determined. Applying the method of Pearson *et al*.^26,27^, the net intensity of the white lines was determined.

Multiple scattering contributions were eliminated by the Fourier-ratio deconvolution method provided by the *EELS Analysis tool* in the DigitalMicrograph Software^23^. However, such contributions are not significant, since the sample thickness (~20 nm) is low compared to the scattering length (~100 nm for MnFe_2_O_4_ according to the *Mean Free Path estimator*^28^). The resulting intensity ratio values are given in Table 1. These values can also be used to determine the valences of some transition metals^29–31^.

**Table 1:** L_3_/L_2_ intensity ratios.

	Core region	Shell region	Difference
Iron	3.69	3.98	0.29/8%
Manganese	4.05	4.09	0.04/1%

The oxygen K edge around 530 eV loss energy shows a complex fingerprint structure as can be seen in Fig. 5b. There is an intense prepeak at 530 eV for the shell region that is absent in the core region. The prepeak arises due to electron transition from the O 1 s shell to the unoccupied O 2p fraction of the O 2p-Fe3d-hybrid orbitals related to the crystal structure and iron oxidation state^30,32^. According to Colliex *et al*.^30^, this supports the presence of the wustite structure in the core and spinel structure in the shell.

2D chemical mappings were recorded for a detailed investigation of the local oxidation state of the metals. Data were recorded in Dual EELS mode to correct slight shifts of the spectrometer or instabilities of the acceleration voltage by using the Zero loss peak (ZLP) position to adjust the zero position of all spectra. The results for the iron mapping are shown in Fig. 4b. Selected spectra (shown in Fig. 4c) from the core and shell region with pronounced features from the Fe^2+^ and Fe^3+^, respectively, were used to fit the single spectra of the spectrum image using the MLLS (multiple linear least square) fit tool from Digital Micrograph. The fit coefficients were used to generate the colour coded map as shown in Fig. 4b.

There is a clear shift of the oxidation state between the iron of the core and the shell region of the particle as already expected from the point spectroscopy from Fig. 5. The shell thickness amounts to ~4 nm with a clearly visible border between the core and the shell. Using the oxygen K edge prepeak intensity confirms the core-shell structure. In contrast, based on the manganese spectra, no change of the oxidation state is observed for the manganese ions from this kind of measurement (not shown).

**Figure 6:**
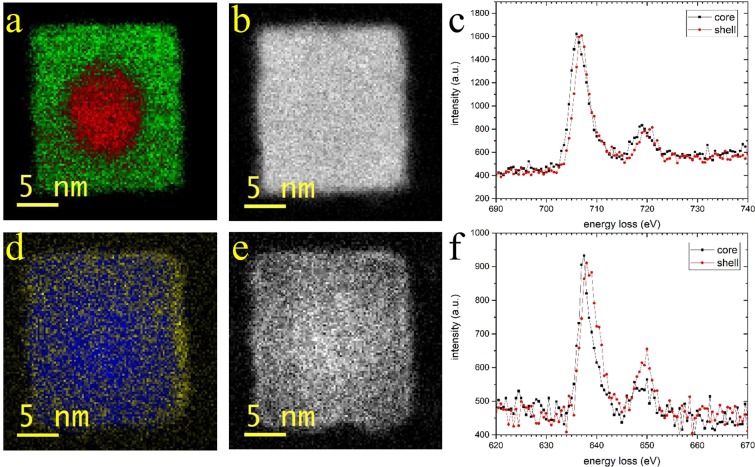
High-resolution analysis of a single Fe/Mn-Oxide particle. (**a**) color coded oxidation state map of the iron. Red: Fe^2+^; green: Fe^3+^. (**b**) iron intensity map (integrated intensity from 702–712 eV after background subtraction). (**c**) iron L_2_, L_3_ edges from core and shell, respectively. (**d**) oxidation state map of the manganese (Blue: Mn^2+^; yellow: Mn^3+^). (**e**) manganese intensity map (integrated from 633–643 eV after background subtraction). (**f**) manganese L_2_, L_3_ edges from core and edge, respectively.

Figure 6 shows a higher spatial resolution EELS map of a single nanoparticle which was recorded to observe further details in the oxidation state distribution of the metal ions. The iron mapping confirms the results inferred from Fig. 4. However, the high resolution measurement shows a thin surface layer (<~1 nm) of manganese ions in a higher oxidation state than in the bulk of the particle.

The samples were stored for approximately 3–6 months under ambient conditions before the EELS measurements were performed. In contrast to the observations of Chen *et al*.^15^ for pure iron oxide nanoparticles prepared in a similar way, our particles are stable against further oxidation for at least this timespan. TEM bright field images show the core-shell contrast even after a storage time of one year.

The Fe:Mn ratio in the particles (core and shell region, respectively) does deviate from the atomic ratios in the educts. The Fe:Mn atomic ratio in the educts was set to 2:1. The measured atomic ratios from EELS and EDS in the core and shell, respectively, are given in Table 2.

**Table 2:** Atomic ratios of Fe and Mn in core and shell, respectively.

	Fe	Mn
EDS, Core	77	23
EELS, Core	76	24
EDS, Shell	80	20
EELS, Shell	79	21

## Discussion

Cubic shaped core-shell Particles with a MnO/FeO core and a Mn_x_Fe_3-x_O_4_-like shell were produced in a one pot synthesis by thermal decomposition of a mixture of the corresponding metal oleate complex precursors Mn(II)-oleate and Fe(III)-oleate, in the presence of Na-oleate as surfactant. The cubic shape is due to the used surfactant, as shown by various groups for pure iron oxide nanoparticles^33–36^. It has the same effect when applied to a Fe-oleate/Mn-oleate mixture. Shape control is due to the fact that the surfactant influences the growth speed in certain crystallographic directions, so that the final crystal shows only specific faces. Here, the cube facets are parallel to the {100} planes. Elemental mapping shows a homogeneous distribution of the iron in the core and shell, while the manganese concentration is slightly higher in the core region. However, this is in contrast to the work of Bodnarchuk *et al*.^20^, who prepared core-shell particles from the same precursors in a similar way, but found the manganese present only in the shell. We can only speculate about the reason for this. There are two different possibilities: the difference can be due to diffusion processes after growth or due to different decomposition behaviour of the oleate precursors during synthesis due to slight differences in the synthesis route. Bodnarchuck used a lower heating rate of 2 °C/min, while here a heating rate of 25 °C/min was applied. According to Kemp *et al*.^37^, and Chang *et al*.^38^, the iron oleate structure is based on a triironoxonium core. It is therefore more complex compared to the Mn^2+^ oleate structure. The oleates may behave different regarding their stability and decomposition performance. Assuming the Mn-oleate to decompose at a higher temperature (i.e. to be more stable) that is reached later in case of a lower heating rate, there already could have formed iron oxide cores before the Mn comes into play. In our case with a higher heating rate, the time until the critical decomposition temperature for the Mn-oelate is reached is too short so that no iron oxide cores have been formed. This could explain the Mn in the core. However, Chang *et al*.^38^ found that the particle formation in case of the decomposition of iron oelate is not based on a classical burst nucleation, but happens continuously, accompanied by a continuous growth of the particles. If this applies to the manganese oleate as well, one would expect both metals to be present in the core of the particles.

Other approaches, using Fe(acac)_3_/Mn(acac)_2_ as precursors to make cubic MnFe_2_O_4_ nanoparticles, have observed both, a core shell structure with separated metal ions^22^ as well as single phase particles^21^. As explanation for the formation of a core-shell structure the large gap in decomposition temperature between Fe(acac)_3_ and Mn(acac)_2_ is given^21^.

On the surface of the particles, sodium was detected by TEM-EDS measurement. Since the only source for sodium is the used surfactant sodium oleate, we conclude that this surfactant sticks quite strong to the surface and could not be removed by the applied cleaning procedure of the particles. Since the mappings reveal a carbon-rich layer around the particles, we assume the oleic acid anions (from the surfactant or precursor, respectively) to be present on the particle surface even after cleaning with hexane/isopropanol. This is also manifested in the very good dispersibility of the particles in organic media like n-hexane, indicating an organic (unipolar) surface.

Furthermore, in TEM images of all samples a typical gap of ~2–3 nm between the particles can be observed. This is attributed to the length of the oleic acid molecule. Since the length of an oleic acid molecule is ~2.4 nm^39^, it can be deduced that there is a strong overlapping of the surfactant molecules of adjacent particles. It should be noted that by secondary electron (SE) images taken together with STEM DF images it was ensured that the adjacent particles from which the gap width was determined were located on the same side of the grid (not shown).

The overall elemental distribution of the metal ions differs only slightly between core and shell (see Table 2). The core-shell contrast in the TEM bright field images must therefore arise mostly due to Bragg contrast. The slight difference in the manganese concentration between the core and shell region, respectively, is a direct result of the phases. However, EELS and EDX quantifications lead to the fact that the Mn:Fe ratio in the particles is lower than expected from the amount of educts. EDS measurements covering larger and thicker regions of the sample result in a Fe:Mn = 2:1 ratio. This leads to the conclusion, that some of the Mn precursor has not decomposed or the Mn is not incorporated in the particles.

The oxidation states of the metals were determined from the EELS measurements. The shift of the Fe L_3_ edge (Fig. 5) indicates a higher oxidation state in the shell region^23,24^. However, such a shift is not only valence specific, but can also result from occupation of different sites in different crystal structures^25^. For the case of iron, Taftø^25^ found a shift of ~1.3 eV between the valence states 2+ and 3+, respectively, while different sites may lead to a shift of only 0.7 eV, so that the absolute shift of the iron L_3_ edge can add up to 2 eV.

The increase of the peak width is assumed to arise due to the presence of Fe^2+^ and Fe^3+^ in the shell, whereas in the core only Fe^2+^ is present. Therefore, it can be concluded that the oxidation state of the iron in the shell region of the particles is higher with respect to the core region, whereas the oxidation state of the manganese seems to be constant for the measured regions except a very thin surface layer.

According to the literature, the L_3_/L_2_ ratio is decreasing for increasing oxidation state for manganese^29^. Iron, however, shows a different behaviour, resulting in higher L_3_/L_2_ ratios for higher oxidation states^30^. This is due to the electron occupancy of the 3d orbitals, being maximal for Fe^2+^ and decreasing for higher oxidation states. The ratios of the white lines confirm the valences found from the peak position measurements. The value of 4.05 and 4.09 for manganese fits almost perfectly to the obtained values from Wang *et al*.^29^ for various Mn^+2^ compounds. The value from the iron, however, does not fit to the literature values. It should be noted that the absolute values given in the literature are varying significantly. Sparrow *et al*.^31^ found a change from 3.3 to 3.6 between FeO and Fe_2_O_3_, whereas Colliex *et al*.^30^ gives values between 3.9 and 4.2. Our value changes from 3.7 to 4.0. This is due to the fact that the absolute values depend strongly on the exact method of background subtraction, peak fitting method and kind of sample. However, the relative change is always found to be around 7–9% of the net intensity what is in good agreement with our measurement (8% change).

The stability of the Mn^2+^ against further oxidation can be explained with its 3d5-configuration, that is also responsible for the stability of the Fe^3+^ state. Since the oxidation state of the Mn in the precursor is 2+, it does not change during the high-temperature synthesis. However, at least some of the iron does change its oxidation state from 3+ in the precursor to 2+ in the final core of the particles. This is consistent with the work of Feld *et al*.^18^, who found a reduction of Fe^3+^ due to oleate intermediates formed during the reaction.

It could not be resolved whether the 3 +iron in the shell region is reduced during synthesis and oxidized again during post-processing and storage or whether it stays in the 3 +state during synthesis. However, the structure is stable under ambient conditions for at least 1 year. Excluding oxygen (<1 ppm) during post-processing and storage of the particles by utilizing a glove-box does not change the observed core-shell structure significantly. This stable core shell structure is in contrast to the observation of Chen *et al*.^15^, who prepared wustite nanoparticles in a similar way and observed a continuous oxidation to the spinel-type iron oxide phases. One difference to our synthesis is the absence of surfactants like Na-oleate. Since we found Na on the surface of the particles even after the cleaning procedure, we assume that there is a protective layer of Na-oleate that may retard the oxidation process under ambient conditions.

Another explanation for the stable core-shell structure could be the mechanism of the oxidation process of wustite. If we assume that the process is ruled by diffusion of iron ions from the core to the shell, it is possible that the process slows down rapidly when a certain shell thickness is reached. From the EELS map shown in Fig. 6 it can be seen that the core got an almost spherical shape. Iron atoms at the faces of the cube have a lower chance to diffuse to the surface than iron atoms at the corners, since the reachable surface area at the corners is higher (1D against 3D situation).

From the prepeak of the oxygen-K edge it can be concluded that the core of the particles consists of a FeO/MnO structure, whereas the shell shows a ferritic structure with Mn^2+^ and Fe^3+^, respectively, forming a spinel like Mn-ferrite structure. However, carbon contamination during the STEM measurements made maps with higher resolution impossible. FFT analysis of the high resolution images confirm these findings concerning the structure.

Other groups observed a similar passivation behaviour for pure iron or wustite nanoparticles with stable core-shell structures^40,41^.

There was no evidence for other iron oxide phases like hematite or even pure iron that could have been formed during disproportionation of FeO as known from bulk material^42^.

## Conclusion

In this work, cubic nanoparticles with about 20 nm edge length were synthesized from a mixture of Fe-oleate and Mn-oleate precursors. For shape control Na-oleate was applied.

The nanoparticles show a core-shell structure with 4 nm shell thickness. High-resolution TEM and EELS measurements reveal the crystal structures and valences of the metals within the particles. In the core region, a FeO/MnO structure is found, with Fe and Mn in the 2+ state. The shell consists of a spinel like Mn-ferrite structure. While the iron shows a higher oxidation state in the shell, the manganese is in the 2+ state within this shell, showing a higher oxidation state only at a very thin (<1 nm) surface layer. Both metals, Fe and Mn, respectively, are distributed within the particles without separation. Mn shows a slight concentration gradient from the core to the shell. The observed core-shell structure is stable against oxidation under ambient conditions for at least 1 year. By EDS, a closed layer of Na can be detected on the surface of the particles, that stems from the used surfactant.

## Data Availability

The data generated during and/or analysed in the current study are available from the corresponding author on reasonable request.
